# An equity dashboard to monitor vaccination coverage

**DOI:** 10.2471/BLT.16.178079

**Published:** 2016-11-21

**Authors:** Catherine Arsenault, Sam Harper, Arijit Nandi, José M Mendoza Rodríguez, Peter M Hansen, Mira Johri

**Affiliations:** a Department of Epidemiology, Biostatistics and Occupational Health, McGill University, 1020 Pine Avenue West, Montreal, Quebec H3A 1A2, Canada.; b Statistics Canada, Ottawa, Canada.; c Gavi, the Vaccine Alliance, Geneva, Switzerland.; d Centre de recherche du Centre Hospitalier de l’Université de Montréal (CRCHUM), Montreal, Canada.

## Abstract

Equity monitoring is a priority for Gavi, the Vaccine Alliance, and for those implementing *The* *2030 agenda for sustainable development*. For its new phase of operations, Gavi reassessed its approach to monitoring equity in vaccination coverage. To help inform this effort, we made a systematic analysis of inequalities in vaccination coverage across 45 Gavi-supported countries and compared results from different measurement approaches. Based on our findings, we formulated recommendations for Gavi’s equity monitoring approach. The approach involved defining the vulnerable populations, choosing appropriate measures to quantify inequalities, and defining equity benchmarks that reflect the ambitions of the sustainable development agenda. In this article, we explain the rationale for the recommendations and for the development of an improved equity monitoring tool. Gavi’s previous approach to measuring equity was the difference in vaccination coverage between a country’s richest and poorest wealth quintiles. In addition to the wealth index, we recommend monitoring other dimensions of vulnerability (maternal education, place of residence, child sex and the multidimensional poverty index). For dimensions with multiple subgroups, measures of inequality that consider information on all subgroups should be used. We also recommend that both absolute and relative measures of inequality be tracked over time. Finally, we propose that equity benchmarks target complete elimination of inequalities. To facilitate equity monitoring, we recommend the use of a data display tool – the equity dashboard – to support decision-making in the sustainable development period. We highlight its key advantages using data from Côte d’Ivoire and Haiti.

## Résumé

Le suivi de l'équité est une priorité pour Gavi, l'Alliance du Vaccin et pour ceux qui mettent en œuvre le *Programme de développement durable à l'horizon 2030*. Dans le cadre de sa nouvelle phase d'opérations, Gavi a repensé son approche relative au suivi de l'équité en matière de couverture vaccinale. Afin de contribuer à cet effort, nous avons réalisé une analyse systématique des inégalités en matière de couverture vaccinale dans 45 pays soutenus par Gavi et comparé les résultats obtenus à partir de différentes méthodes de mesure. Nous nous sommes appuyés sur nos conclusions pour formuler des recommandations concernant l'approche adoptée par Gavi pour suivre l'équité. Cette approche impliquait de définir les populations vulnérables, de choisir des mesures appropriées pour quantifier les inégalités et d’établir des critères en matière d'équité qui reflètent les ambitions du programme de développement durable. Dans le présent article, nous expliquons la raison d'être de nos recommandations et le but de l'élaboration d'un meilleur outil de suivi de l'équité. L'approche précédemment utilisée par Gavi pour mesurer l'équité consistait à calculer la différence en matière de couverture vaccinale entre les quintiles de richesse les plus élevés et les plus bas d'un pays. Nous recommandons de suivre des dimensions de la vulnérabilité (éducation maternelle, lieu de résidence, sexe des enfants et indice de pauvreté multidimensionnelle) autres que l'indice de richesse. Lorsqu'une dimension inclut divers sous-groupes, il convient d'utiliser des mesures de l'inégalité prenant en compte les informations relatives à tous les sous-groupes. Nous conseillons également de suivre les mesures absolues mais aussi relatives d'inégalité au fil du temps. Enfin, nous suggérons que les critères en matière d'équité visent l'élimination complète des inégalités. Afin de faciliter le suivi de l'équité, nous recommandons l'utilisation d'un outil d'affichage de données – le tableau de bord de l'équité – pour favoriser la prise de décision dans le cadre du programme de développement durable. Nous mettons en avant les principaux avantages de cet outil à l'aide de données provenant de Côte d'Ivoire et d'Haïti.

## Resumen

La supervisión de la equidad es una prioridad para la Gavi, la Vaccine Alliance y para los que implementan la Agenda 2030 para el Desarrollo Sostenible. Para su nueva fase de operaciones, la Gavi reevaluó su enfoque para supervisar la equidad en la cobertura de vacunación. Para ayudar a informar este esfuerzo, se realizó un análisis sistemático de desigualdades en la cobertura de vacunación en 45 países apoyados por la Gavi y se compararon los resultados desde distintos enfoques de medición. En base a los resultados, se formularon recomendaciones para el enfoque de supervisión de equidad de la Gavi. El enfoque implicó la definición de las poblaciones vulnerables, la selección de las medidas adecuadas para cuantificar las desigualdades y la definición de las referencias de equidad que reflejan las ambiciones de la agencia de desarrollo sostenible. En este artículo, se explican los motivos de las recomendaciones y el desarrollo de una herramienta mejorada de supervisión de la equidad. El anterior enfoque de la Gavi para la medición de la equidad era la diferencia de la cobertura de vacunación entre los sectores demográficos más ricos y más pobres de un país. Además del índice patrimonial, se recomienda supervisar otras dimensiones de vulnerabilidad (educación de la madre, lugar de residencia, sexo de los niños y el índice de pobreza multidimensional). Para las dimensiones con múltiples subgrupos, deberían utilizarse medidas de desigualdad que tienen en cuenta información acerca de todos los subgrupos. También se recomienda que, con el paso del tiempo, se haga un seguimiento tanto de la medida de desigualdad absoluta como relativa. Por último, se propone que las referencias de equidad tengan como objetivo la eliminación completa de la desigualdad. Para facilitar la supervisión de la equidad, se recomienda utilizar una herramienta de indicación de datos (el tablero de equidad) para apoyar la toma de decisiones durante el periodo de desarrollo sostenible. Se destacan sus ventajas básicas utilizando datos de Côte d’Ivoire y de Haití.

## ملخص

تعد عملية رصد تحقيق المساواة في التغطية التطعيمية من أهم أولويات التحالف العالمي للقاحات والتحصين (Gavi) لإنتاج الأمصال والقائمين على تطبيق جدول أعمال التنمية المستدامة لعام 2030. وقام التحالف العالمي للقاحات والتحصين بإعادة تقييم استراتيجيته للمرحلة الجديدة لضمان تحقيق المساواة في التغطية التطعيمية. وللمساعدة في تحقيق تلك المهمة قمنا بإجراء تحليل منهجي للفروقات في التغطية التطعيمية بين 45 دولة تدعم الاتحاد ومقارنة نتائج القياس المختلفة. كما قمنا بصياغة توصيات لاستراتيجية الاتحاد المتعلقة برصد عملية المساواة في التغطية التطعيمية من واقع النتائج التي توصلنا إليها. وتشمل تلك الاستراتيجية تحديد الشرائح الاجتماعية الهشة واتخاذ الإجراءات المناسبة وقياس الفروقات في التغطية التطعيمية وتحديد معايير المساواة التي تعكس طموحات جدول أعمال التنمية المستدامة. وسوف نقوم ضمن هذا المقال بشرح الأساس المنطقي لتلك التوصيات وتطوير أداة رصد تحقيق المساواة في التغطية التطعيمية. كانت الاستراتيجية السابقة التي كان الاتحاد ينتهجها تقوم على أساس الاختلافات في التغطية التطعيمية بين الفئات الخمسية السكانية الغنية والفقيرة داخل الدولة الواحدة. ونوصي برصد الزوايا الأخرى الهشة والضعيفة (تعليم الأمهات ومكان الإقامة وجنس الطفل ومؤشر الفقر متعدد الجوانب) إلى جانب مؤشر الثروة. وينبغي الاعتماد على مؤشرات قياس عدم المساواة التي تراعي المعلومات المتعلقة بجميع الفئات الفرعية وذلك لمتابعة الجوانب التي تضم مجموعات ثانوية متعددة. كما نوصي أيضًا برصد مؤشرات قياس عدم المساواة المطلقة والنسبية بمرور الوقت. وأخيرًا نقترح أن تهدف معايير المساواة إلى إنهاء الفروقات في التغطية التطعيمية بشكل تام. ولتسهيل عملية رصد تحقيق المساواة في التغطية التطعيمية، فإننا نوصي باستخدام أداة عرض البيانات المعروفة باسم "لوحة متابعة الاستفادة من الحقوق" لدعم اتخاذ القرارات خلال فترة التنمية المستدامة. ونقوم بتسليط الضوء على مزاياها الرئيسية باستخدام البيانات المأخوذة من كوت ديفوار وهايتي.

## 摘要

公平性监测是全球疫苗免疫联盟 (Gavi) 以及实施* 2030 年可持续发展议程*国家的重点工作。 对于其新运作阶段,全球疫苗免疫联盟重新评估了其监测疫苗接种覆盖率公平性的方法。 为了帮助开展此次评估,我们系统地分析了 45 个全球疫苗免疫联盟支持国家疫苗接种覆盖率的不公平性,并对比了采用不同测量方法所取得的结果。 基于我们的发现,我们编制了全球疫苗免疫联盟公平性监测方法的制定建议。 方法包括定义弱势群体、选择适当的方式量化不公平性以及定义符合可持续发展议程目标的公平性基准。 本文中,我们对这些建议及开发改良性公平性检测工具的根本原因进行了阐述。 全球疫苗免疫联盟先前的公平性监测方法是测量一个国家财富五分位数中最富有人群和最贫穷人群之间的疫苗接种覆盖率差距。 除了财富指数,我们还推荐对其他弱势群体衡量维度(母亲受教育程度、居住地、儿童性别以及多维贫穷指数)进行监测。 对于多群组衡量维度,应使用将所有分组信息考虑在内的不公平性检测措施。 同时,我们还建议对绝对和相对不公平性检测措施进行实时追踪。 最后,我们提议公平性基准应针对彻底消除不公平性而制定。 为了推进公平性监测,我们推荐使用一款数据显示工具——公平性监测仪表盘——以辅助可持续发展阶段的决策制定。 我们使用来自科特迪瓦和海地的数据突着重说明了其关键优势。

## Резюме

Контролирование равномерности вакцинации — это приоритет для GAVI (Global Alliance for Vaccines and Immunisation — Глобальный альянс по вакцинам и иммунизации) и для всех, кто участвует в реализации *Повестки дня в области устойчивого развития на период до 2030 года* ООН. В свете новой фазы работы GAVI была проведена переоценка подхода к отслеживанию равномерности охвата вакцинацией. Для информационной поддержки об этих мероприятиях нами был проведен систематический анализ неравномерности охвата вакцинацией по 45 странам, поддерживаемым GAVI, и проведено сравнение результатов, полученных с использованием разных подходов. На основе наших данных были сформулированы рекомендации для GAVI по подходу к контролю равномерности охвата. Этот подход включает определение уязвимых групп населения, выбор подходящих мер для количественной оценки неравномерности и определение эталона равномерности, который отражает устремления программы по устойчивому развитию. В данной статье объясняется рациональность этих рекомендаций и развития более совершенного механизма контроля равномерности. Предыдущий подход GAVI для измерения равномерности был основан на различии охвата вакцинацией между странами, попадающими в богатейшие и беднейшие квинтили по параметрам благополучия. В дополнение к этому параметру в качестве показателя благополучия мы предлагаем использовать другие параметры уязвимости (образование у матерей, место жительства, сексуальную активность несовершеннолетних и многомерный индекс бедности). Для параметров со множественными подгруппами следует использовать показатели неравномерности, которые включают информацию по всем подгруппам. Мы также рекомендуем как абсолютные, так и относительные показатели неравномерности, которые должны отслеживаться во времени. В завершение мы предлагаем считать, что целью эталонов равномерности является полное устранение неравномерности. Для облегчения контроля равномерности рекомендуется использовать данные инструмента отображения — панели равномерности, чтобы облегчить принятие решений во время устойчивого развития. Его ключевые преимущества освещаются с использованием данных Кот-д’Ивуара и Гаити.

## Introduction

*The* *2030 agenda for sustainable development* calls upon the international community to prioritize the needs and rights of the most vulnerable, so that no one is left behind.^1^ Some population groups in low- and middle-income countries continue to be systematically missed by lifesaving health interventions, such as childhood immunization.^2^ Determining effective ways to measure and monitor progress in addressing social exclusion is a priority for those involved in implementing the sustainable development goals (SDGs), including for Gavi, the Vaccine Alliance.

Gavi’s strategy for 2016–2020 includes a focus on equity in vaccination coverage.^3^ The 2016–2020 strategy has catalysed a re-examination of Gavi’s approach to monitoring equity. In an earlier phase of its operations, Gavi’s principal indicator of equity was the difference in coverage between a country’s richest and poorest wealth quintiles.^4^ This indicator is also used by many other international organizations and has been widely applied in health equity analyses.^5^

Several questions have emerged in relation to Gavi’s equity monitoring for the 2016–2020 period. Is the earlier approach, based largely on a single dimension and measure, sufficient to monitor progress in addressing inequalities in the SDG period? Is the wealth quintile the most appropriate indicator to assess inequalities in access to vaccines? Does the coverage gap between rich and poor capture the full extent of inequality and allow meaningful international comparisons?

To answer these questions, we conducted a systematic analysis of inequalities in childhood vaccination coverage based on different measurement approaches, using the most recent (as of May 2015) demographic and health surveys (DHS) in 45 Gavi-supported countries.^6^ Our findings enabled us to formulate recommendations on the equity monitoring approach that would best support policies and practices tailored to the most vulnerable. In particular, we propose the use of a data display tool – the equity dashboard – to monitor inequalities and support decision-making in the SDG period. The purpose of this article is to explain the rationale for the recommendations and for developing the equity dashboard. We illustrate the key advantages of the dashboard using examples from specific countries.

## Dimensions of vulnerability

A first consideration in equity monitoring is how to define the vulnerable; that is, to determine which population characteristics describe those who are left behind. For example, SDG17 highlights income, sex, age, race, ethnicity, migratory status, disability and geographical location as important sources of vulnerability. This calls for a disaggregation of data according to these factors and other characteristics relevant to national contexts.^7^ For Gavi, the indicators selected must be applicable globally and must allow comparisons across countries. Therefore, although they may be highly relevant in specific settings, certain characteristics such as race, ethnicity or religion will be impractical for the global monitoring of inequalities since they are measured differently across countries and reflect local social structures.^8^

We used a framework from the World Health Organization’s (WHO) Commission on Social Determinants of Health^9^ to review potential determinants of child health inequities. From this framework, we identified five indicators and composite measures: (i) the wealth index; (ii) maternal education; (iii) place of residence; (iv) child sex; and (v) the multidimensional poverty index. These were chosen as they describe population groups at risk of differential vaccination coverage; are available in household surveys in low- and middle-income countries, such as DHS and multiple indicator cluster surveys (MICS); and are measured in a similar way across countries.

Although no single indicator is ideal, each has a claim to be relevant to equity monitoring. The wealth index is the indicator most commonly used in equity monitoring. It measures socioeconomic position and attempts to capture the material aspects of living conditions.^10^ Often called the asset index, it is based on a household’s ownership of assets, its access to safe drinking water and the quality of sanitation and housing. It is initially calculated as a continuous index and often split into quintiles that allow for straightforward comparisons between the richest and poorest quintiles within a country.^11^ Nonetheless, the wealth index has an important limitation for cross-country comparisons, as it only measures relative socioeconomic position, i.e. specific to a given country. The bottom (poorest) quintile in a middle-income country may be better off than the top (richest) quintile in a low-income country.^12^

Parental education, another indicator of socioeconomic position, is related to health and vaccination status through multiple pathways.^9^ Educated mothers generally have better knowledge of good medical practices, a higher social status and greater autonomy and decision-making power, making them more able to communicate with and access health services.^13^ Determining whether mothers with little or no education are able to access vaccination services should be a part of Gavi’s equity monitoring strategy. In addition, in contrast to wealth quintiles, education levels can be better translated between low- and higher-income countries.^10^ However, education has an added complexity for inequality measurement. Unlike wealth quintiles, which divide population samples into five groups, each containing about 20% of all households, the number of individuals in each category of education can be uneven. For example, there may be few (or no) mothers without any formal education in middle-income countries, and few mothers who completed secondary school in low-income country samples.

Geographical location is another important determinant of child health inequities.^9^ To compare geographical inequalities across countries, an indicator for urban or rural residence can be used. Policy-makers should consider whether vaccination services are reaching rural areas. However, urban and rural subgroups are broad categories that could conceal some of the geographical inequality present. For example, relatively wealthy urban areas and urban slums with mass deprivation may be collapsed into a single category.^8^ A further breakdown by smaller geographical units at national levels might offer a more precise picture of inequalities.

Although progress has been made in the past decades towards reducing gender inequalities in access to health resources worldwide, differences between the sexes in child health outcomes remain in some countries.^14^ Determining whether girls and boys have equal access to immunization services is crucial from a human rights perspective.

Conceptually, another important source of inequity relates to the concurrence of key health determinants that place some children simultaneously at greater risk of acquiring vaccine-preventable diseases and at lower probability of surmounting them. Unimmunized children are often differentially exposed to further health risks, such as inadequate nutritional intake, poor water and sanitation, indoor air pollution and overcrowding, all of which increase the risk of infectious diseases.^9^^,^^15^ Unimmunized children are also more vulnerable to poor health outcomes once infected, as their parents often lack health knowledge and access to other preventive interventions and medical care.^15^^,^^16^ These systematic, overlapping deprivations are particularly important dimensions of vulnerability.

Finally, the multidimensional poverty index, reported annually since 2010 in the United Nations Development Programme’s *Human development report*, is a measure of overlapping deprivations at the household level in developing countries. The index addresses the concept of systematic exposure to multiple burdens, an approach which is neglected by traditional measures of socioeconomic position (such as those that focus only on asset deprivation). The multidimensional poverty index shows not only who is poor but also how they are poor: what simultaneous disadvantages they experience.^17^ The index is a continuous indicator calculated by the weighted sum of deprivations in 10 indicators of health, education and living standards that are commonly available in household surveys (adult household members’ years of schooling, child school attendance, child mortality, nutrition, cooking fuel, sanitation, water, electricity, house-flooring material and assets).^17^ By including indicators for child mortality and nutrition, the index directly identifies children at higher risk of adverse health outcomes and hence at highest priority for vaccination from an equity standpoint. Nonetheless, a child’s nutritional status can itself be affected by infection and lack of vaccination. We believe that the index is particularly valuable for descriptive surveillance of vaccination coverage and for orienting policy. However, like all other indicators presented here, its association with vaccination coverage should not be interpreted in causal terms. It has been widely recommended that equity monitoring include several diverse definitions of vulnerability.^5^^,^^8^^,^^18^^,^^19^ The WHO Health Equity Monitor database, for example, reports estimates for several maternal and child health outcomes disaggregated by wealth quintiles, maternal education, sex and area of residence (urban or rural), derived from the DHS and MICS.^20^ Similarly, the Countdown to 2030 collaboration disaggregates coverage of health interventions by wealth quintiles, maternal education, sex, area of residence and country region.^21^ In addition to these indicators, we believe that the multidimensional poverty index, as well as its individual components of health, education and living standards, is particularly relevant to monitoring inequalities in vaccination coverage.

To formulate recommendations based on empirical findings, we measured inequalities in vaccination coverage across 45 Gavi-supported countries and compared results from different measurement approaches (Arsenault C et al., McGill University, unpublished data, 2016). Using the most recent DHS in each country, we measured inequalities in the receipt of the third dose of diphtheria–tetanus–pertussis-containing vaccine (DTP3) and measles-containing vaccines according to the five selected indicators. Coverage with DTP3 and measles-containing vaccines are widely accepted as standard indicators of how well a country’s immunization system is performing.^22^ Although the five indicators were correlated, they differed in their ability to identify vulnerable groups in specific contexts. We found the largest inequalities according to the multidimensional poverty index, maternal education and the wealth index. Inequalities by place of residence and child sex were lower on average, but revealed important inequalities in specific countries. Many equity analyses include only measures of wealth-based inequality. However, the wealth index does not by itself reflect the full complexities of social disadvantage. Our analysis showed that while a country may have equitable coverage according to the wealth index, inequalities could be present in other dimensions that may be equally relevant to policy-making.

## Measures of inequality

Equity monitoring entails another important consideration: the choice of appropriate measures to quantify inequalities. The absolute and relative coverage gaps are the measures most commonly used. The absolute coverage gap is typically applied to wealth quintiles and consists of measuring the difference in coverage between the richest (Q5) and poorest (Q1) quintiles (Q5 minus Q1). The relative coverage gap is the ratio of coverage between the richest and poorest quintiles (Q5 divided by Q1). When indicators have only two subgroups, such as child sex or urban versus rural residence, the absolute and relative coverage gaps are the most straightforward way to measure inequality. However, when the whole population is not included in the two subgroups compared, the coverage gaps have limitations. For example, when comparing Q5 and Q1, quintiles 2, 3 and 4 are ignored. This can conceal important heterogeneity and may provide a limited view of the inequalities present across the entire range of social groups.^23^^,^^24^ This measure is also impractical for multiple education levels or continuous indicators such as the multidimensional poverty index which require arbitrary subgroups to be defined.

Alternative measures of inequality may overcome these limitations. The slope index of inequality and the relative index of inequality have been recommended for multiple subgroups or continuous indicators because they consider information on all subgroups, summarize inequality across the whole distribution and reflect the direction of the gradient in health.^23^^–^^27^ The slope and relative indices of inequality can be obtained by regressing the child’s vaccination status on her or his cumulative rank in the population’s socioeconomic distribution.^23^^,^^25^

In our empirical analysis, we first tested the use of both the coverage gap (absolute and relative) and the slope and relative indices of inequality to assess education, multidimensional poverty and wealth-related inequalities in vaccination coverage. We found that the coverage gaps were more likely to generate imprecise estimates if there were few individuals in the extreme categories of education or of the multidimensional poverty index, this made comparisons of the level of inequality across countries more difficult. In contrast, the slope and relative indices of inequality were less likely to be affected by sampling error and produced more reliable country comparisons. Although they involve additional complexity and assumptions, the slope and relative indices of inequality may be better suited than coverage gap measures for international comparisons of the levels of inequality.^23^^,^^28^

We also found that comparisons of the level of inequality across countries differed substantially whether absolute or relative measures were used. The fact that absolute and relative measures can lead to different conclusions when comparing inequalities across time and place has been frequently reported.^27^^,^^29^^–^^32^ To obtain a complete picture of inequalities and a reliable measurement of progress over time, we designed the equity dashboard to include both absolute and relative measures.

## Equity benchmarks

Equity monitoring aims to promote social justice and the realization of human rights, and these concepts can be reflected in the definition of benchmarks and targets. In its 2011–2015 strategy period, Gavi established a minimum equity benchmark requiring that DTP3 coverage in the poorest wealth quintile be no more than 20 percentage points lower than coverage in the richest wealth quintile.^4^ This benchmark was set empirically to provide a realistic and attainable target for Gavi-supported countries. However, acceptance of up to a 20% points difference in coverage between the richest and poorest is difficult to justify from an ethical standpoint.

We propose a new target for the SDG period, inspired by the Indian economist and philosopher Amartya Sen’s vision of human development as the freedom to achieve well-being.^33^ Following Sen, we view health as valuable both in itself, and for its contribution to expanding people’s effective freedoms or opportunities to undertake the activities that they find valuable and to live the lives that they find meaningful.^34^ Because vaccination is a key determinant of the potential for health over the life course, equality in vaccination is crucial to achieving true equality of opportunity in the capability for health.

We therefore argue that the appropriate target for immunization equity should be equal coverage. We interpret this to mean that there should be no meaningful inequalities across social groups: a judgement that must incorporate both quantitative metrics and knowledge of the characteristics of the country and sample. In practice, this requires testing whether the magnitude of inequality is statistically distinguishable from zero. To avoid confusing moral significance with statistical significance, this benchmark requires adequate sample sizes to achieve precise estimates. Precision benchmarks could also be established by defining acceptable ratios of 95% confidence limits.^35^ An equal coverage benchmark is conceptually defensible in that it reflects a commitment to the moral equality of persons and fulfilment of the human right to health. It is also realistic and attainable. Among the 45 Gavi-supported countries we analysed, 38% (17 countries) had already met the benchmark for equal coverage across the wealth index (Arsenault C et al., McGill University, unpublished data, 2016).

## Equity dashboard

Based partly on early results from our analysis, Gavi has lowered the equity benchmark to 10 percentage points and added indicators related to geographical equity and maternal education. This added complexity in Gavi’s strategy for 2016–2020 raises new challenges, prompting us to propose the use of an equity dashboard for more effective monitoring of equity in vaccination coverage in the SDG period.

**Fig. 1:**
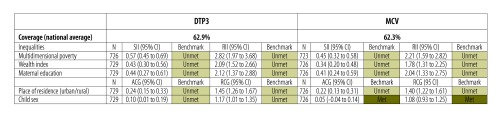
**Example of an equity dashboard for monitoring vaccination coverage, Côte d’Ivoire, 2011–2012**

ACG: absolute coverage gap; CI: confidence interval; DTP3: third dose of diphtheria–tetanus–pertussis-containing vaccine; MCV: measles-containing vaccines; N: number of children aged 12–23 months; RCG: relative coverage gap; RII: relative index of inequality; SII: slope index of inequality.

Note: Benchmarks are met when the 95% CI of the inequality estimate includes the null value (absolute 0, relative 1) and unmet when the 95% CI excludes the null value.

Source: Data for this analysis (Arsenault C et al., McGill University, unpublished data, 2016) were extracted from the Côte d’Ivoire demographic and health survey 2011–2012.^36^

**Fig. 2:**
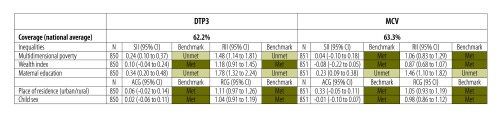
**Example of an equity dashboard for monitoring vaccination coverage, Haiti, 2012**

ACG: absolute coverage gap; CI: confidence interval; DTP3: third dose of diphtheria–tetanus–pertussis-containing vaccine; MCV: measles-containing vaccines; N: number of children aged 12–23 months; RCG: relative coverage gap; RII: relative index of inequality; SII: slope index of inequality.

Note: Benchmarks are met when the 95% CI of the inequality estimate includes the null value (absolute 0, relative 1) and unmet when the 95% CI excludes the null value.

Source: Data for this analysis (Arsenault C et al., McGill University, unpublished data, 2016) were extracted from the Haiti demographic and health survey 2012.^37^

To illustrate the advantages of this approach we present the vaccination equity dashboards for two countries that have similar levels of national vaccination coverage but very different equity profiles. The equity dashboards for Côte d’Ivoire and Haiti (Fig. 1 and Fig. 2) were based on data from children aged 12–23 months from the most recent DHS in 2011–2012.^36^^,^^37^ These show the national average coverage for DTP3 and measles-containing vaccines, and the inequalities in children’s receipt of these vaccines across the five selected indicators of vulnerability. The slope and relative indices of inequality were used for indicators that were continuous or in multiple ordered subgroups: maternal education (no education, incomplete primary, complete primary, incomplete secondary, complete secondary, higher education attended), the wealth index and the multidimensional poverty index. The absolute and relative coverage gaps were used for indicators with two subgroups: place of residence (urban versus rural) and child sex (male versus female). Equity benchmarks are marked in the dashboard as met if the 95% confidence interval (CI) of the inequality estimate includes the null value, or unmet if the 95% CI excludes the null value.

In Côte d’Ivoire (Fig. 1), the equity dashboard shows inequalities in DTP3 coverage across all five dimensions, including child sex. DTP3 coverage is 10 percentage points higher among boys than among girls (slope index of inequality: 0.10; 95% CI: 0.01 to 0.19). The inequality estimates revealed by the multidimensional poverty index are also substantially higher than those revealed by wealth and education. National coverage with DTP3 and measles-containing vaccines are 62.9% and 62.3% respectively.

In the case of Haiti (Fig. 2), the dashboard reveals virtually no inequalities by children’s sex or place of residence or between the richest and poorest households according to the wealth index. Gavi’s initial approach required that the absolute coverage gap between the richest and poorest wealth quintiles be no more than 20 percentage points. As this coverage gap was 10 percentage points (95% CI: –0.05 to 0.25) in the 2012 DHS,^37^ Gavi would have considered Haiti as having achieved equitable vaccination coverage. However, the equity dashboard reveals substantial inequalities by maternal education and multidimensional poverty. For example, DTP3 coverage is on average 34 percentage points higher (slope index of inequality: 0.34; 95% CI: 0.20 to 0.48) among children of mothers at the top versus the bottom of the distribution of maternal education, and measles-containing vaccine coverage is 23 percentage points higher (slope index of inequality: 0.23; 95% CI: 0.09 to 0.38). Similarly, the least deprived children are 1.5 times (relative index of inequality: 1.48; 95% CI: 1.14 to1.81) more likely to receive DTP3 than children deprived in most or all of the multidimensional poverty index components. These measures imply systematic associations between low vaccination coverage and, respectively, low education and high multidimensional poverty. More importantly from a policy perspective, relying solely on the wealth index would fail to identify these vulnerable groups. In addition, the dashboard highlights that, although Haiti meets the equity benchmarks across three dimensions (wealth index, place of residence and child sex), national coverage with DTP3 and measles-containing vaccines remain at only 62.2% and 63.3%. In addition to equity benchmarks, national coverage benchmarks could be established to target policy efforts.

Comparing the two dashboards also demonstrates that, although national coverage with the two vaccines is almost identical in both countries, the magnitude of inequalities is considerably higher in Côte d’Ivoire compared with Haiti.

The equity dashboard offers a single snapshot of a country’s overall achievement and inequalities across multiple dimensions of social exclusion and vulnerability. We propose that the dashboard improves equity monitoring and can serve as a decision support tool for policy development. It may also facilitate conversation and be readily useable by those without technical expertise in inequality measurement. By providing a comprehensive and standardized analysis of inequalities, the dashboard also facilitates comparisons across countries (and between different vaccines). As new data become available, the dashboard should also include historical trends of inequalities in each dimension to track changes over time. A third criterion could then be introduced for the equity benchmarks, represented by yellow cells in the dashboard, to indicate whether progress was being made in reducing inequalities.

## Conclusion

Given current global efforts to achieve universal health coverage (SDG3), reduce inequalities (SDG10) and increase the availability of disaggregated data (SDG17), countries and development partners must make equity monitoring a priority.^38^ Gavi and development partners should develop equity dashboards tailored to their specific policy needs. An equity dashboard may also be a useful decision-support tool for other stakeholders seeking to monitor equity in coverage of other health services. This type of monitoring may help determine where and why inequalities arise and ensure that policies are successful in improving the health of the most vulnerable.^38^
